# Aberrant Left Colic Artery and Accessory Right Colic Artery: A Case Report and Surgical Implications

**DOI:** 10.7759/cureus.40594

**Published:** 2023-06-18

**Authors:** Axel Lichtenberg, So Jung Kim, Logan Rogers, Joel Jung, Megha Rajput, Camri Rawls, April N Alford, Korey Walter, Kamal A Abouzaid, Ahmad Imam

**Affiliations:** 1 Department of Anatomical Sciences, William Carey University College of Osteopathic Medicine, Hattiesburg, USA

**Keywords:** gastrointestinal surgical anatomy, surgical vascular complications, vascular anomaly, vascular imaging, laparoscopic right hemicolectomy, cadaver case report, accessory right colic artery, aberrant left colic artery, superior mesenteric arteries

## Abstract

During a routine dissection of the abdominal region in our department of anatomy’s dissection laboratory, we found two variations of the vasculature of the gastrointestinal tract within the greater peritoneal and retroperitoneal compartments: an aberrant left colic artery (LCA) and an accessory right colic artery (RCA). The aberrant LCA originates from a common trunk (CT) that arises from the superior mesenteric artery instead of the inferior mesenteric artery. The CT continues for a short distance and terminates by dividing into a middle colic artery and an accessory RCA. The aberrant LCA and accessory RCA had abnormal courses; therefore, they are vulnerable to injury during surgical procedures of the region. Hence, a thorough knowledge of vascular variations is required to avoid potential complications.

## Introduction

According to Tandler’s hypothesis [1], in early human embryos, four primitive splanchnic arteries originate from the dorsal aorta and are linked to a ventral longitudinal anastomosis. As development progresses, the primitive arteries develop into three arteries, corresponding to the celiac trunk, the superior mesenteric artery (SMA), and the inferior mesenteric artery (IMA). This is followed by the disappearance of the longitudinal anastomosis. Deviation in the regression of this primitive arterial system is responsible for various variations [1,2].

The arterial supply for the bowels is from the celiac trunk, SMA, and IMA. The IMA, the major artery of the hindgut, normally branches into the left colic artery (LCA), the sigmoid arteries, and the superior rectal artery. The LCA is the main source of blood supply to the distal third of the transverse colon and proximal descending colon through its ascending branch (AB). The descending branch (DB) of the LCA supplies the distal descending colon and anastomoses with the superior sigmoid artery [3]. In addition, the IMA provides collateral circulation to the SMA, the major artery of the midgut, through the marginal artery. The SMA gives off the inferior pancreaticoduodenal artery, ileocolic artery (ICA), right colic artery (RCA), and middle colic artery (MCA). The RCA and the MCA are the branches that supply the ascending colon and the proximal two-thirds of the transverse colon, respectively [4].

## Case presentation

**Figure 1:**
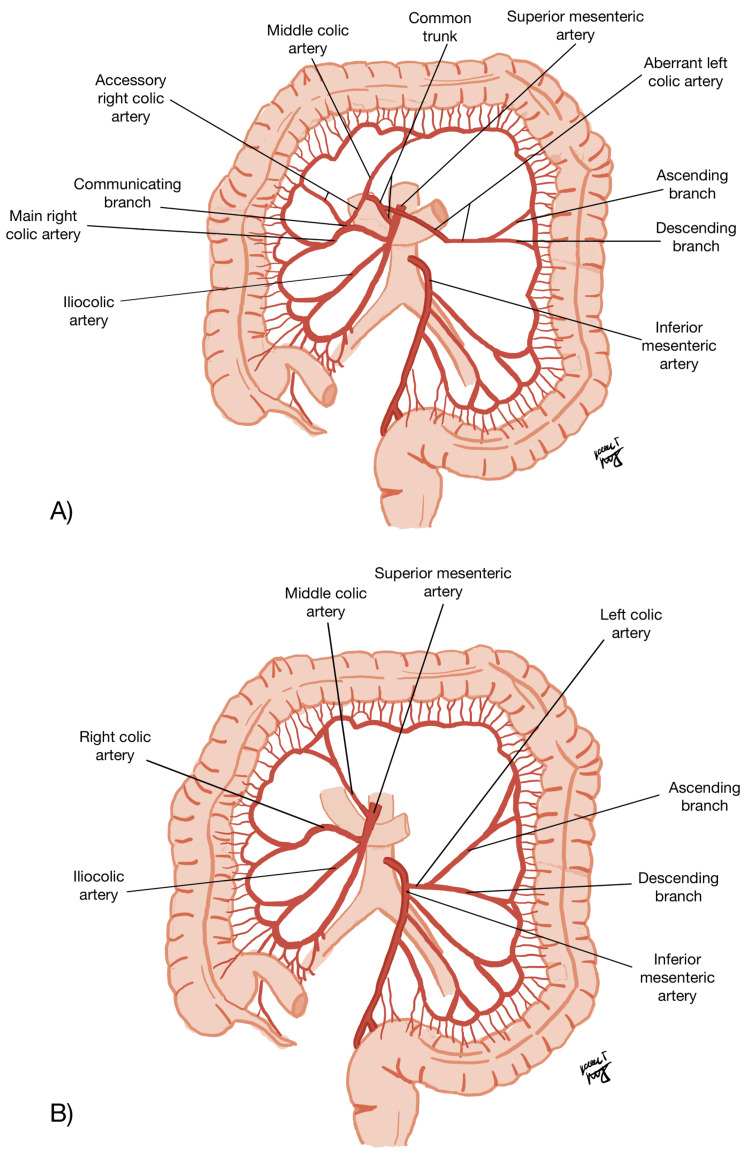
A schematic representation of the arterial variation. The arterial variation seen in this case (A), and normal bowel arteries for comparison (B). Image credits: Karen Lichtenberg

**Figure 2:**
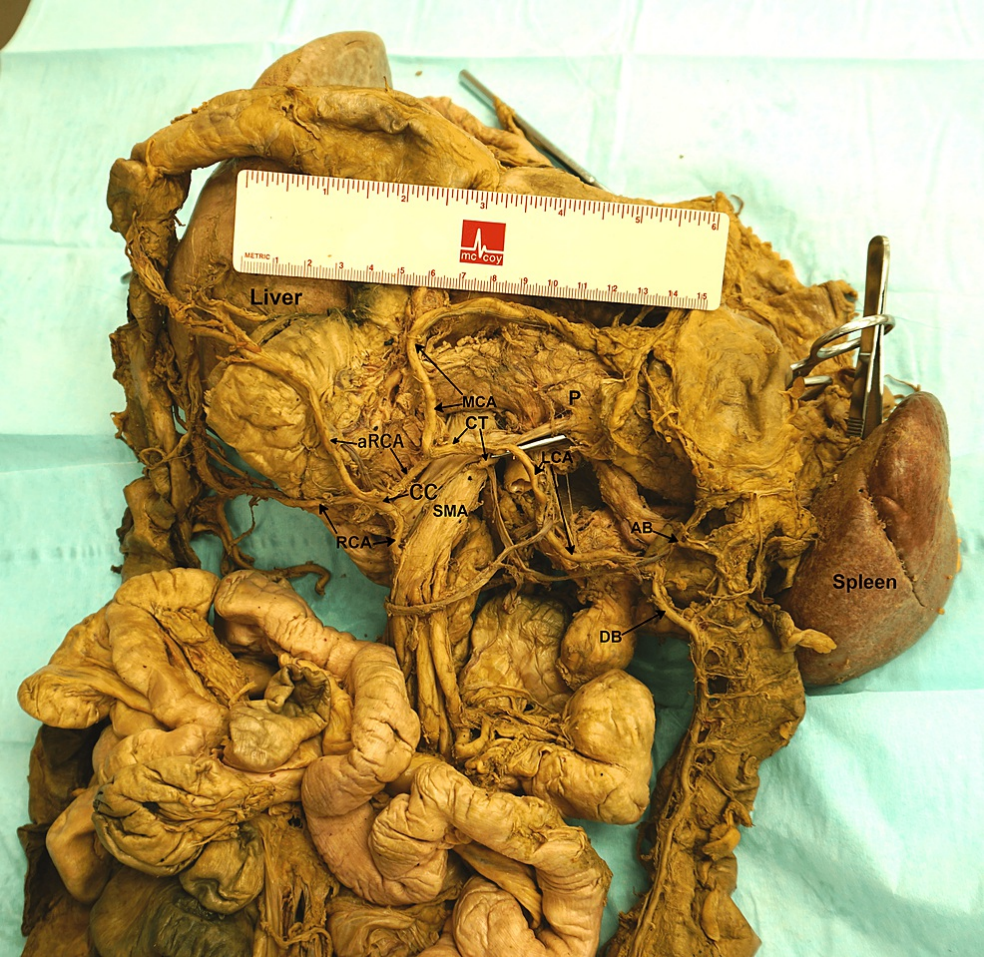
The branching pattern of the SMA. RCA = right colic artery; aRCA = accessory right colic artery; CC = communicating branch; MCA = middle colic artery; LCA = aberrant left colic artery; CT = common trunk; SMA = superior mesenteric artery; AB = ascending branch of the left colic artery; DB = descending branch of the left colic artery; P = pancreas.

**Figure 3:**
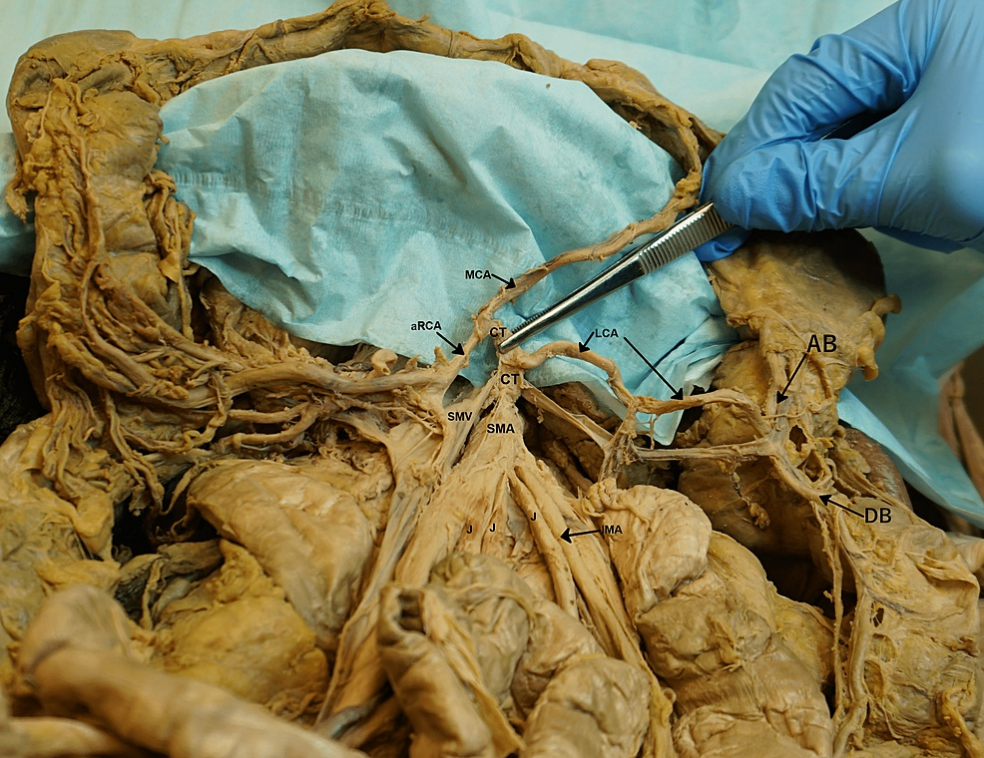
Branches of the common trunk: the aberrant LCA, MCA, and an aRCA. aRCA = accessory right colic artery; MCA = middle colic artery; LCA = aberrant left colic artery; CT = common trunk; SMA = superior mesenteric artery; SMV = superior mesenteric vein; J = jejunal branches of superior mesenteric artery; IMA = inferior mesenteric artery; AB = ascending branch of the left colic artery; DB = descending branch of the left colic artery.

**Figure 4:**
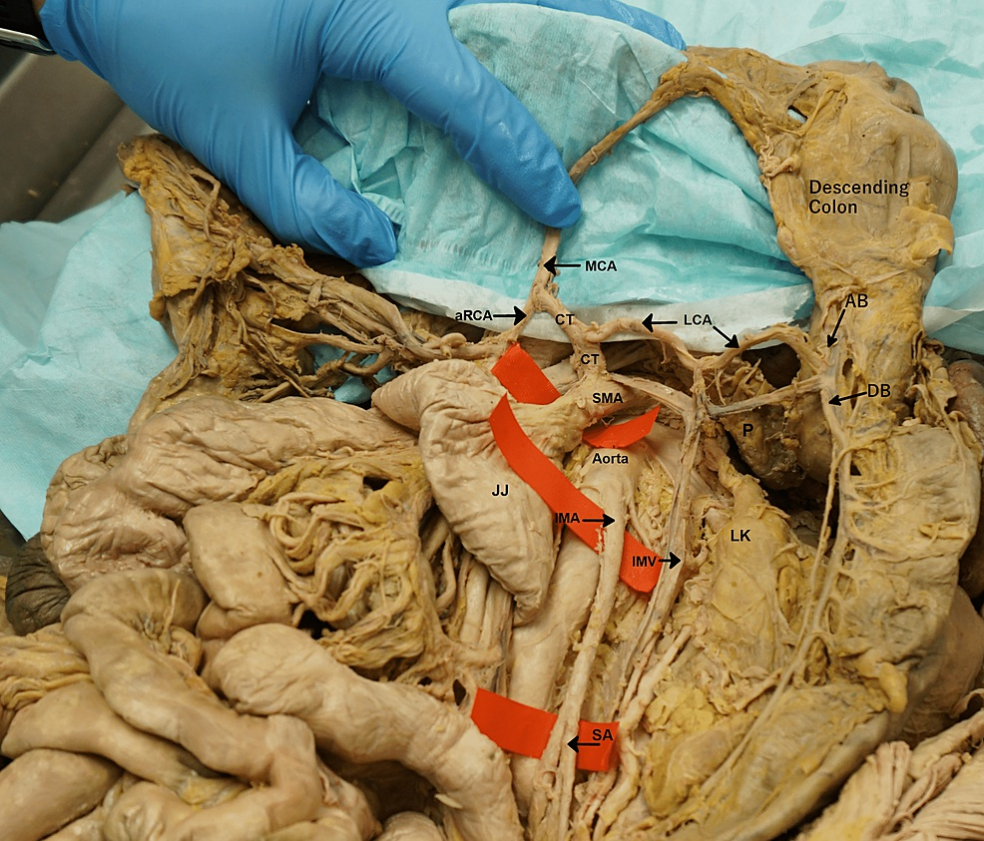
The aberrant LCA as it courses inferior to the body of the pancreas to divide into its AB and DB, which join the marginal artery to supply the descending colon and splenic flexure. RCA = right colic artery; aRCA = accessory right colic artery; MCA = middle colic artery; LCA = aberrant left colic artery; AB = ascending branch of the left colic artery; DB = descending branch of the left colic artery; CT = common trunk; SMA = superior mesenteric artery; SMV = superior mesenteric vein; JJ = jejunum; J = jejunal branches of the superior mesenteric artery; IMA = inferior mesenteric artery; IMV = inferior mesenteric vein; SA = sigmoidal arteries; LK = left kidney; P = pancreas.

**Figure 5:**
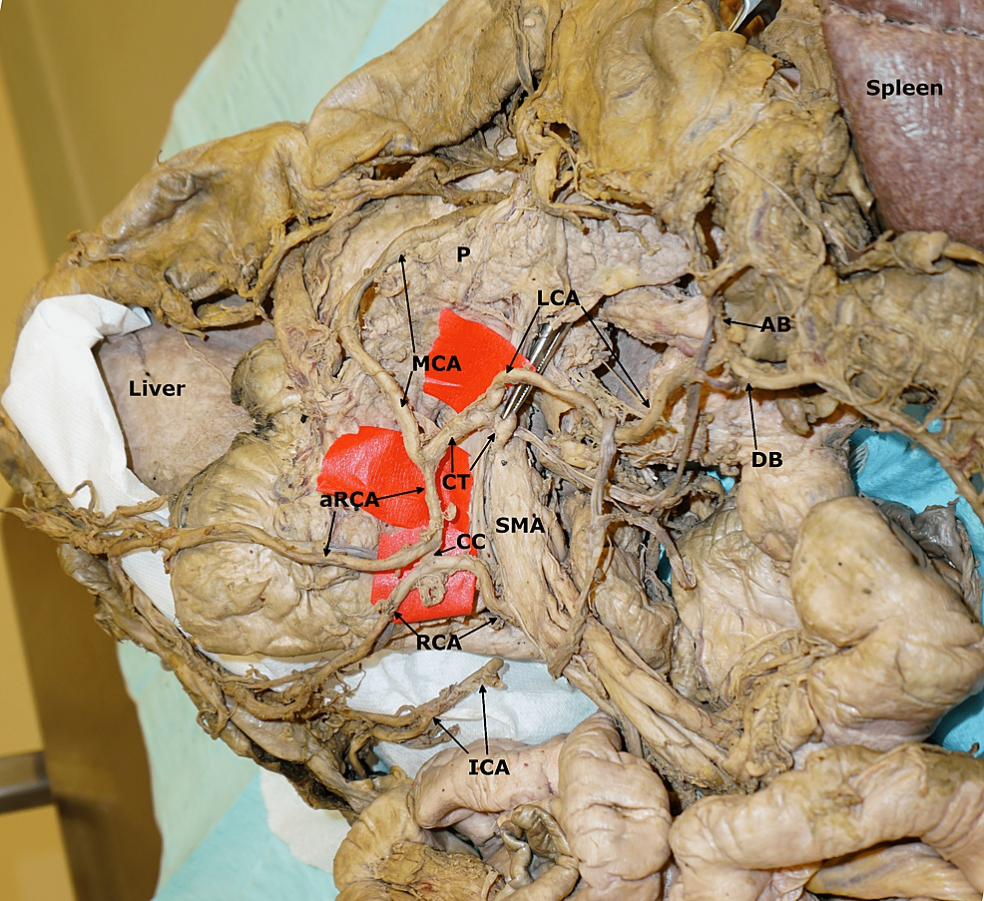
The courses of the main RCA and accessory RCA inferior to the head of the pancreas. RCA = right colic artery; aRCA = accessory right colic artery; CC = communicating branch; MCA = middle colic artery; LCA = aberrant left colic artery; CT = common trunk; SMA = superior mesenteric artery; AB = ascending branch of the left colic artery; DB = descending branch of the left colic artery; P = pancreas; ICA = ileocolic artery (cut during dissection).

We report a rare variation in the normal branching pattern of the SMA found by medical students during cadaveric dissection (Figure 1). The variation was observed in an 87-year-old Caucasian female cadaver received from the University of Southern Alabama Anatomical Gift Program who, based on the death certificate, had died from respiratory failure. The branches of the SMA were carefully dissected and their origin, course, and termination were studied. The SMA gives off its first branch, the common trunk (CT), approximately 2 cm inferior to the proximal segment of the body of the pancreas (Figure 2). In total, the CT gives rise to three other branches: the aberrant LCA, MCA, and an accessory RCA (Figure 3). The CT first branches to form the aberrant LCA, which courses inferior to the body of the pancreas to divide into its AB and DB and joins the marginal artery to supply the descending colon and splenic flexure (Figure 4). The CT continues for a short distance and terminates to form the MCA and accessory RCA. The MCA courses superiorly and supplies the transverse colon. The accessory RCA courses to the right, inferior to the head of the pancreas, and has a small communicating branch (CC) that anastomoses with the main RCA (Figure 5). The accessory RCA then continues to the right and supplies the midsection of the ascending colon and hepatic flexure. The main RCA branches directly off the SMA and supplies the proximal middle section of the ascending colon superior to the cecum. The ICA branches off the SMA at its usual place of origin and supplies the cecum and the distal ileum (Figure 5).

It is worth noting that there are some documented cases where the aberrant LCA branches off the SMA rather than the IMA, but this phenomenon is still relatively unexplored and not well understood. This variation, including an aberrant LCA and the addition of an accessory RCA, to our knowledge, has not been reported before. Understanding vascular variations is necessary to avoid jeopardizing blood supply during surgical resection procedures.

## Discussion

Due to the multitude of collateral routes of the mesenteric arteries, planned vascular clamping during surgical procedures is crucial to avoid vascular injury and necrosis. Understanding the variations of the colic arteries before surgical procedures, such as laparoscopic colectomies and resection of specific organs, is critical to avoid preventable damage to nearby viscera. For instance, in a Chinese study of 582 individuals, 2% of patients coming in for esophageal grafting using the colon had an aberrant LCA that did not branch from the IMA [5]. Lack of consideration for the aberrant LCA poses potential complications. Hence, the colon may no longer be used as a safe substitutive graft due to the risk of jeopardizing the blood supply of the remaining segment of the colon [6]. In addition, aberrant LCAs have been observed to branch directly from the SMA in cadavers [7,8] and from the SMA in a live patient [6]. The retroperitoneal location of the aberrant LCA is near other various major vessels such as the inferior vena cava and the abdominal aorta. Thus, this area is particularly susceptible to injury during pancreatic surgeries since surgeons do not expect the aberrant LCA artery in the vicinity [7]. Therefore, prior knowledge of the vasculature in this area and potential anomalies is necessary to minimize surgical complications.

Preoperative vasculature imaging is crucial, especially for laparoscopies due to their narrow field of vision. The impact of three-dimensional imaging of mesenteric vasculature (from computer tomography angiography) on the performance of 112 laparoscopic colectomies or rectal resections, showed a statistically significant reduction in operative time, and lower rates of intraoperative and postoperative complications from errors identifying mesenteric vessels when the surgeon was able to view the three-dimensional reconstructions before and during the surgery [9]. Thus, knowledge of mesenteric vasculature has significant surgical implications and preoperative vascular imaging may be required.

In a previous study [10], the variant branching patterns of the SMA were comprehensively classified into four main categories. Pattern I is described as the independent origin of the three main branches of the SMA. Pattern II is subdivided into three sub-patterns based on the common trunks of origin: pattern IIa (common trunk between RCA and MCA), pattern IIb (common trunk between RCA and ICA), and pattern IIc (common trunk for all three main branches). Pattern III is the absence of the RCA. Pattern IV is the presence of accessory RCAs originating from the SMA. However, the specific branching pattern reported in this study, which includes a common trunk for the MCA, accessory RCA, and aberrant LCA, along with a communicating branch between the accessory RCA and main RCA, cannot be classified using the aforementioned classification system. This variation pattern shares similarities with pattern IIa (common trunk for MCA and RCA) and pattern IV (presence of accessory RCAs). However, pattern IIa does not include the presence of the aberrant LCA, and pattern IV does not include communication branches between RCAs. Thus, the pattern observed in this case does not fit the existing classification system for variant branching patterns of the SMA.

Although there is no current research on this specific aberrant LCA and accessory RCA variation, in one study of 607 patients, over a third of participants had variations in mesenteric arteries [11]. During a right hemicolectomy for malignancies, the surgeons ligate and cut the ICA and RCA blood vessels, along with the lymph nodes in the area [12]. However, because of the possibility of an accessory RCA, it would be crucial to also identify and ligate this artery and remove the lymph nodes around it to prevent the spread of cancer.

## Conclusions

It has been reported that variations of LCA and RCA are not uncommon; however, to our knowledge, the vascular variants reported in this paper do not appear in the literature. The proximity of the aberrant LCA and accessory RCA to abdominal organs and major abdominal vessels warrants further consideration when performing surgical procedures as they pertain to postoperative complications. Therefore, knowledge of not only the common and systemic course of the mesenteric arterial supply is necessary, but also vascular variations in the relevant area. The various anomalies presented in this report also emphasize the importance of proper vascular imaging prior to abdominal surgeries. In conclusion, the possibility of encountering these variations during surgery may warrant the necessity for thorough preoperative radiologic examinations and interpretations.

## References

[1] Fahmy D, Sadek H (2015). A case of absent celiac trunk: case report and review of the literature. Egypt J Radiol Nucl Med.

[2] Yi SQ, Li J, Terayama H, Naito M, Iimura A, Itoh M (2008). A rare case of inferior mesenteric artery arising from the superior mesenteric artery, with a review of the review of the literature. Surg Radiol Anat.

[3] Skinner D, Wehrle CJ, Van Fossen K (2022). Anatomy, Abdomen and Pelvis: Inferior Mesenteric Artery. Anatomy, Abdomen, and Pelvis.

[4] Shaikh H, Wehrle CJ, Khorasani-Zadeh A (2022). Anatomy, Abdomen and Pelvis: Superior Mesenteric Artery. Anatomy, Abdomen, and Pelvis.

[5] Cheng BC, Chang S, Huang J (2006). Surgical anatomy of the colic vessels in Chinese and its influence on the operation of esophageal replacement with colon. (Article in Chinese). Zhonghua Yi Xue Za Zhi.

[6] Christiena A, Kapil N, Ansari I, Ps S, Kannan N (2021). Aberrant left colic artery and its surgical implications. Cureus.

[7] Nayak S, Shetty S, Sirasanagandla S, Aithal A, Shanthakumar S (2013). Anomalous origin and vulnerable course of left colic artery in relation to the pancreas—a case report. Forensic Med Anat Res.

[8] Mishra V, Barua M, Singh B, Sharm V (2018). Variant origin and course of the left colic artery: an embryological perspective. Int J Anat Var.

[9] Mari FS, Nigri G, Pancaldi A (2013). Role of CT angiography with three-dimensional reconstruction of mesenteric vessels in laparoscopic colorectal resections: a randomized controlled trial. Surg Endosc.

[10] Gamo E, Jiménez C, Pallares E, Simón C, Valderrama F, Sañudo JR, Arrazola J (2016). The superior mesenteric artery and the variations of the colic patterns. A new anatomical and radiological classification of the colic arteries. Surg Radiol Anat.

[11] Farghadani M, Momeni M, Hekmatnia A, Momeni F, Baradaran Mahdavi MM (2016). Anatomical variation of celiac axis, superior mesenteric artery, and hepatic artery: evaluation with multidetector computed tomography angiography. J Res Med Sci.

[12] Mitchell BG, Mandava N (2022). Hemicolectomy. https://www.ncbi.nlm.nih.gov/books/NBK555924/.

